# Omics-based decoding of molecular and metabolic crosstalk in the skin barrier ecosystem

**DOI:** 10.1038/s41418-025-01648-8

**Published:** 2026-01-08

**Authors:** Luca Elettrico, Gabriele Piacenti, Chiara Levra Levron, Osamu Ansai, Alessandro Croce, Carlotta Duval, Valentina Proserpio, Giacomo Donati

**Affiliations:** 1 Department of Life Sciences and Systems Biology, University of Turin, Torino, Italy; 2 Molecular Biotechnology Center “Guido Tarone”, University of Turin, Torino, Italy; 3 Italian Institute for Genomic Medicine, Candiolo (TO), Italy

**Keywords:** Inflammation, Microbiology, Gene expression

## Abstract

Skin homeostasis depends on interactions between epithelial cells and the microbiome mediated by molecular and biochemical factors. Perturbations of this interplay are linked to inflammatory disorders, including wound healing and cancer. While research has mainly illuminated shifts in microbial community composition, novel computational approaches are starting to reveal the host-microbe functional interactome in the cutaneous ecosystem. In this review, we specifically focus on known molecular and metabolic mechanisms linking skin epithelial cells and microorganisms in health and disease. Additionally, we summarise computational tools available to investigate these interactions integrating omics data. Furthermore, we present potential applications of this functional crosstalk to advance therapies targeting skin pathologies. Finally, we propose a comparative interactomics approach to envision the existence of ecological memories in the skin ecosystem, in parallel with the one described in the gut, hypothesising a link between epithelial and microbial memories in barrier tissues.

## Facts

- Mounting evidence from low–microbial-biomass epithelia such as the skin indicates that altered microbe–keratinocyte crosstalk functionally contributes to inflammatory diseases.
- While skin-associated bacteria are challenging to visualize, emerging omics technologies facilitate integrated studies that reveal their molecular crosstalk and overcome the limitations of prior separate analyses.
- Incoming computational tools allow to decipher the skin microbiome composition and its interactions with keratinocytes to achieve a system-level resolution by integrating omics data, network analysis and AI-based approaches.
- Comparative interactomics and omic data suggest potential links between epithelial and microbial memories in barrier ecosystems, including skin.
- New tools will reveal epithelial ecological niches and cross-kingdom interactions, guiding the discovery of novel therapeutic targets for skin diseases.

## Skin microbiome and its ecological niches

Mammalian skin, the body’s largest organ, prevents water loss, regulates temperature, and protects against physical, chemical and microbial insults. It is composed of three layers: epidermis, dermis and hypodermis [1]. Epidermis, the outermost layer, is a stratified epithelium consisting of keratinocytes, the most abundant cell component, and other cell types, including melanocytes and Langerhans dendritic cells. Underneath epidermis, the dermis provides pliability, elasticity, and tensile strength to skin, safeguarding the body from mechanical injury. It accommodates two epidermal appendages: the pilosebaceous unit, composed of hair follicles (HF) and sebaceous glands, and the sweat glands. The skin harbors diverse ecological niches that house a heterogeneity of host cellular constituents [2] and a plethora of microorganisms, including bacteria, fungi, viruses, archaea, and mites, collectively forming what we know as the skin microbiome [3, 4]. The composition of skin microbial communities shifts across different regions of the body. For instance, face, chest and back skin are rich in HF and sebaceous glands that secrete sebum, a lipid-rich substrate promoting the growth of lipophilic microorganisms, primarily *Cutibacterium* bacteria and *Malassezia* fungi [3]. In contrast, moist regions like axillae are characterised by numerous sweat glands and are predominantly inhabited by *Staphylococcus* spp. and *Corynebacterium* spp [3]. Dry regions, including palms, have the lowest microbial abundance but the greatest diversity, with prominent populations of *Cutibacterium*, *Corynebacterium*, and *Streptococcus* species [3]. Thus, the skin bacterial and fungal communities are shaped by physiological and environmental features unique to each niche, including the arrays of metabolites that are released in the cutaneous microenvironment. Mammalian skin is also inhabited by viruses that infect host cells, forming part of the microbiome, specifically referred to as the virome. Among others, bacteriophages are key components of the skin microbiome, especially those that target dominant bacterial species such as *Cutibacterium*, *Corynebacterium*, and *Staphylococcus spp* [5]. Although viruses are usually regarded as harmful, several studies suggest that a commensal viral community also exists on healthy skin [5], similar to certain bacteria known for contributing to skin function by protecting against external threats such as pathogens. The most common viral populations in normal human skin are *Papillomaviridae*, *Adenoviridae*, *Anelloviridae*, *Circoviridae*, *Herpesviridae* and *Polyomaviridae* [6]. However, the dynamics and anatomical variations of the skin virome, as well as its changes in response to pathological conditions, remain to be thoroughly studied. At each site, all microbial populations coexist in a dynamic equilibrium throughout lifetime driven by a multitude of endogenous and external variables; indeed skin cells produce metabolites and antimicrobial molecules that limit the overgrowth of other species and help protect the host from pathogens [7]. Moreover, the microbiome can enhance epithelial barrier homeostasis by utilizing quorum sensing mechanisms to inhibit the production of harmful toxins by pathogens [8]. Additionally, recent studies have emphasized the importance of interactions between skin commensals and the immune system [9]. Importantly, these interactions are critical for the maturation of both innate and adaptive immunity, particularly during early life [10].

Despite recent advances in decoding overall skin microbiome composition, our understanding of microbial localisation at the niche level is currently limited due to technical constraints. Few studies have attempted to dissect sub-compartment microbial community structures by imaging techniques and sequencing strategies, revealing a region-specific bacteriome in HFs [11] and the presence of bacterial products in deeper layers such as the dermis [12]. However, whether microbes colonising subepidermal compartments are live or dead is still debated. Additionally, these works have not delved into the crosstalk between microbes and surrounding host cells. Thus, the field of skin microbiome would greatly benefit from technological advances allowing to visualise single microbes at microniche level in vivo in order to extend knowledge on their actual localisation and interaction with host cells.

## Deregulated molecular mechanisms of host–microbe crosstalk in pathological contexts and their applications

### Interactions in homeostasis and cutaneous inflammatory disorders

The epidermal-microbe crosstalk has mainly been analysed with a focused and hypothesis-driven cellular and molecular perspective. More recently, the use of unbiased omic approaches has expanded our knowledge of the microbiome structure and alterations upon disease states. Although the combination of the two approaches is powerful, the former methods alone provided a large amount of data that we will highlight later in this review. In particular, we will emphasize that the skin is a complex ecosystem in which regulation of the interactions between host cells and resident microbes determines the shift from homeostatic balance to aging or disease.

In contrast with the gut, the skin is a dry, acidic, lipid-rich environment, and therefore shows low microbial biomass [13]. Nevertheless, resident microbes are critical for cutaneous homeostasis, particularly by supporting the skin’s essential barrier functions, preventing water loss and assaults by external agents [14]. While layers of tightly packed differentiated keratinocytes represent the physical component of this barrier, the microbiota is crucial for its integrity and function. This role is mediated by the aryl hydrocarbon receptors (AHRs) expressed by keratinocytes. Metabolites produced by skin commensals activate AHRs, thus sustaining epidermal cell differentiation. Indeed, the restoration of the microbiota-AHR axis could ameliorate skin barrier repair in murine disease models characterised by epidermal barrier impairment [15]. In parallel, the microbiome also supports the lipid components of the skin barrier. For example, *Staphylococcus epidermidis* secretes sphingomyelinase, an enzyme needed to produce ceramide, which prevents dehydration [16]. Concerning the chemical nature of skin barrier, skin commensals can digest dead keratinocytes and other waste to convert them into several types of fatty acids. These molecules modulate skin surface pH making it slightly acidic, thus contributing to the formation of the permeability barrier and creating an environment that limits pathogens growth [17]. On the other hand, keratinocytes can modulate the skin microflora, even at the level of specific epidermal niches such as the HF [18].

**Fig. 1:**
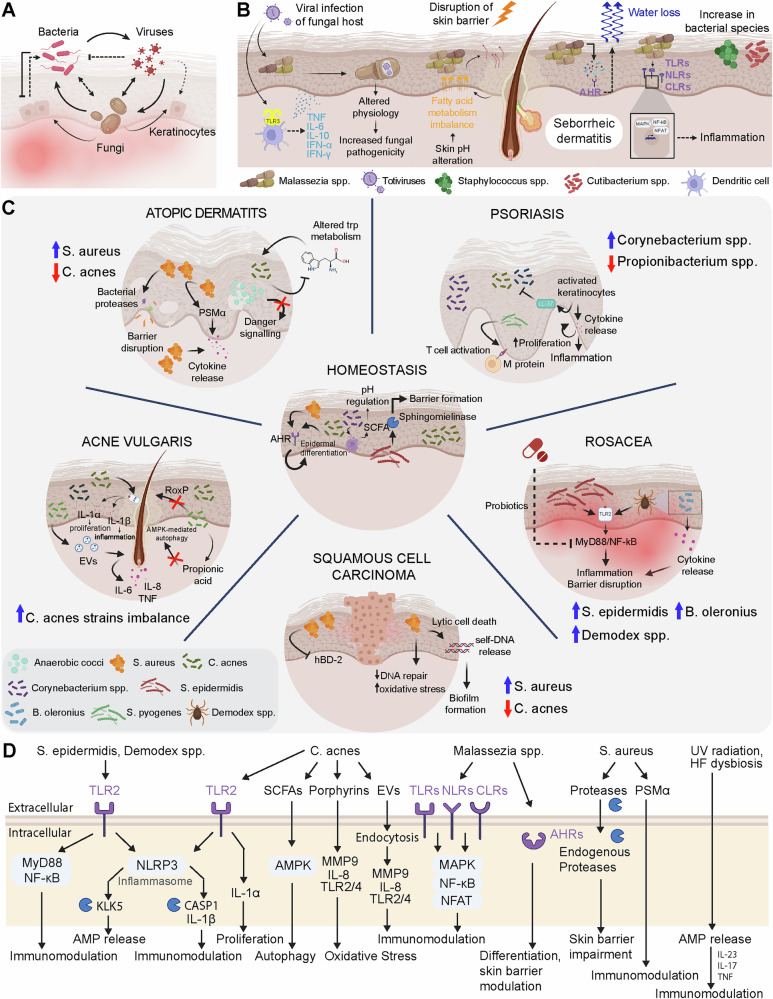
Epidermis-microbiome molecular and metabolic crosstalk in skin inflammatory disorders. **A** Schematic diagram showing the complex network of molecular and metabolic interactions among the components of an inflamed cutaneous ecosystem. **B** Molecular and biochemical crosstalk between keratinocytes and microbes in seborrheic dermatitis. Three major components of the skin microbiome are represented: viruses, bacteria and fungi. **C** Graphical summary reporting key interactions between skin-resident bacteria and keratinocytes in distinct cutaneous inflammatory diseases. Shifts in microbiome composition with respect to homeostasis are also indicated. (**D**) Diagram of key signaling pathways that determine microbial influence on keratinocytes behavior and functions. The draft of the figure was created with BioRender.com. Abbreviations. TLR Toll-like Receptors, MyD88 myeloid differentiation primary response 88, NF-κB nuclear factor-κB, KLK5 kallikrein 5, AMP anti-microbial peptides, NLRP3 NOD-, LRR- and pyrin domain-containing protein 3, IL interleukin, AMPK 5’ AMP-activated protein kinase, SCFAs short-chain fatty acids, MMP9 Matrix metalloproteinase-9, EVs extracellular vesicles, TNF Tumor Necrosis Factor, NLRs NOD-like Receptors, CLRs C-type Lectin Receptors, MAPK mitogen-activated protein kinase, NFAT nuclear factor of activated T cell, AHRs aryl hydrocarbon receptors, PSMα phenol-soluble modulin alpha, UV ultraviolet, HF hair follicle, IFN interferon.

Given the importance of skin microbial balance in maintaining tissue homeostasis, it was not unexpected that alterations in microbiome diversity have been implicated in several skin pathological conditions, including inflammatory disorders (Fig. 1A). Indeed, several studies have gathered evidence of the intricate interactions between skin-resident microbes and host cells in shaping a disease-promoting environment [19, 20]. Notably, inflammation-related pathways seem to be involved in the vast majority of these interactions [21–23]. Nonetheless, other biological processes such as metabolism [24] and oxidative stress [25] can take part in this crosstalk. Following pioneer studies focusing on the gut microbiome both in health and disease [26], there is a clear path towards integrating omic data from skin microbial communities. However, despite the increasing amount of evidence being accumulated for specific skin microbes with a one-to-one approach, the molecular mechanisms linking microorganisms with stem and differentiated cells in skin diseases still have to be elucidated in more detail at an integrated cross-kingdom level.

Below we present host-microbe molecular interactions in the most commonly studied skin inflammatory conditions, providing also a summary of the main molecular cascades triggered by the microbiome in keratinocytes (Fig. 1B-D).

#### Seborrheic dermatitis (SD)

It is a chronic inflammatory skin disease characterised by the accumulation of oily scales and dandruff in areas rich in sebaceous glands. It is associated with cutaneous dysbiosis, mainly involving the fungal commensal *Malassezia spp*. [27] (Fig. 1B). A critical factor that determines the beneficial or detrimental effects of these fungi in skin is the pH. Indeed, this parameter can deeply influence the growth and metabolic activity of *Malassezia spp* [28]. In physiological state, the skin pH is around 6, thus creating a slightly acidic environment. Under these conditions, *M.furfur*, *M.japonica* and *M.yamatoensis* release lipids that counteract the IL-6-mediated pro-inflammatory environment, therefore preserving tissue homeostasis. In SD, the pH is altered and *Malassezia spp*. display an impaired metabolism. In particular, *Malassezia* fungi colonize lipid-rich skin sites where they metabolize fatty acids producing oleic acid, a type of unsaturated fatty acid that causes fatty acid imbalance, thus disrupting the skin barrier function. Moreover, *Malassezia spp*. can directly interact with epidermal cells by activating inflammatory pathways and their effectors such as mitogen-activated protein kinase (MAPK), NF–κB, and nuclear factor of activated T cell (NFAT) [19]. In addition, *Malassezia* metabolites are able to activate AHRs in keratinocytes, thus altering skin barrier integrity [29]. The functional role of *Malassezia spp*. in SD is further confirmed by effective treatments based on probiotics and antifungal compounds, which were shown to improve symptoms in SD patients [30]. Alterations of the abundance of other microorganisms in SD-affected skin are also reported. For instance, in SD bacteria such as *Staphylococcus* and *Propionibacterium* spp. and fungi including *Candida*, *Aspergillus*, and *Filobasidium* are enriched [31], even though their causal role remains to be clarified. Finally, dsRNA Totiviruses that infect fungal cells have been isolated from clinical specimens containing *Malassezia* species, suggesting a role of this interkingdom interaction in promoting fungal pathogenicity [32].

#### Acne vulgaris

Acne is a common inflammatory skin disease affecting the pilosebaceous unit. It results from a combination of increased sebum production by sebaceous glands and hyperkeratinization, with a known role for transcription factor GATA6 deregulation in its pathogenesis [33]. Due to its high prevalence in human populations, acne pathogenesis has also been widely studied in relationship with skin microbiome perturbations (Fig. 1C). Notably, *Cutibacterium acnes* (formerly known as *Propionibacterium acnes*) is described as a driving force leading to acne [19]. Indeed, by activating toll-like receptor 2 (TLR2), it promotes keratinocyte proliferation via interleukin-1 alpha (IL-1α), resulting in comedogenesis (i.e. cornification of HF infundibulum). Moreover, TLR2 pathway also activates NOD-, LRR- and pyrin domain-containing protein 3 (NLRP3) inflammasome and caspase 1, driving the secretion of IL–1β, thus causing inflammation. However, metagenomic studies carried out on healthy *vs* acneic skin suggest that the imbalance in *C.acnes* strains diversity, and not in the absolute density of *C.acnes*, is responsible for acne pathogenesis [34]. Indeed, *C.acnes* is the most abundant commensal bacterium in healthy HFs, where it contributes to regulating homeostasis. For instance, *C.acnes* secretes propionic acid, a short-chain fatty acid that induces autophagy in keratinocytes via 5’ AMP-activated protein kinase (AMPK) activation and mitochondrial alteration [35]. This mechanism protects against pathogenic microorganisms. Additionally, *C.acnes* produces an antioxidant enzyme, called radical oxygenase of *P.acnes* (RoxP), which decreases oxidative damage in keratinocytes [25]. A study performed on several skin cell types in vitro revealed that pathogenic *C.acnes* strains release extracellular vesicles (EVs) with a specific cargo that activates inflammation‑related factors such as IL‑8, IL‑6 and tumor necrosis factor (TNF) [36]. On the contrary, other *C.acnes* phylotypes produce EVs that contain protective proteins. Therefore, depending on the specific *C.acnes* subtype, the effect on skin cells might be beneficial or detrimental.

#### Hidradenitis suppurativa (HS)

Also known as *acne inversa*, it is a chronic inflammatory disorder affecting moist skin areas rich in apocrine glands [18]. HS arises upon HF occlusion caused by hyperkeratosis, followed by inflammation, leading to the formation of subcutaneous nodules and abscesses. Its pathogenesis is thought to be related to altered immune responses and auto-inflammatory events [37]. However, several studies point out that there might be a close association between follicular dysbiosis and HS [18]. Particularly, alterations in microbial species have been observed in HS lesions such as a decrease of *Propionibacterium spp*. [18]. In addition, HS patients show a dominance of *S.aureus* and anaerobic bacteria belonging to *Corynebacterium*, *Porphyromonas*, and *Peptoniphilus spp*. [38, 39]. Increased colonization by these microbes promotes secretion of antimicrobial peptides (AMPs) like psoriasin, LL-37 and β-defensin 2, which have a pro-inflammatory action, thus initiating inflammation. These molecules recruit innate immune cells to HFs, that secrete TNF and activate nuclear factor-κB (NF-κB). It has also been reported that a set of mutations in the γ-secretase genes is associated with HS in cases of familial transmission. Functional impairment of γ-secretase has been demonstrated to alter HF development and function, which might result in follicular occlusion and HS pathogenesis through its impact on keratinocyte differentiation and proliferation, and T cells impairment [40]. Another important interaction between epidermal cells and skin microbiome in HS pathogenesis is described at the metabolic level [24]. Specifically, increased degradation of tryptophan (trp) by skin microbes seems correlated with the early stages of the disease, as its depletion promotes colonization by trp-independent *S.aureus*. On the other hand, reduced trp levels lead to a lower production of AHR agonists by trp-dependent skin commensals. Therefore, the epidermal AHR pathway is less active and cannot counteract chronic inflammation. HS mouse models for studying this process in vivo are still limited, as early efforts reproduced only some clinical features in mouse xenograft [41].

#### Rosacea

It is a chronic inflammatory skin disease characterised by facial redness. Its pathogenesis is associated with alterations in the innate immune system and neurovascular dysregulation [42]. Bacterial products are recognised by higher levels of TLR2 in keratinocytes [43], leading to the activation of the inflammasome, ultimately resulting in the increased secretion of kallikrein 5 (KLK5), a serine protease that promotes the maturation of antimicrobial peptides such as cathelicidin. This causes an inflammatory response mediated by type I interferons and a shift in skin microbiome and promotes disease progression (Fig. 1C). For instance, higher abundance of *S.epidermidis* is linked to rosacea-inflamed skin [20]. Moreover, rosacea-affected skin is associated with higher infestation by *Demodex folliculorum* and *Demodex brevis*, two commensal parasitic mites [20]. Mechanistically, increased local skin temperature - a common symptom of rosacea patients - favors mobility and survival of these arthropods. These microbes can exacerbate the disruption of skin barrier function via the above-mentioned TLR2 pathway, activated by cell membrane components of *Demodex* mites. Interestingly, the bacterium Bacillus oleronius, isolated from Demodex mites infesting the HFs of rosacea patients, has been shown to stimulate an inflammatory response in the skin, thus contributing to disease flares. Furthermore, gut microbiome involvement in rosacea needs to be clarified, despite several studies proposing a correlation between *Helicobacter pylori* and rosacea as treatment with fecal-derived oral probiotics ameliorated rosacea symptoms in a rosacea mouse model [43].

#### Atopic dermatitis (AD)

It is a chronic inflammatory skin disease characterised by relapsing pruritic flares, which strongly affect patients’ life. A clear relationship was demonstrated between AD onset and severity, and alterations of cutaneous flora (Fig. 1C). Specifically, microbial diversity is reduced in untreated flares [20]. Of note, AD pathogenesis has commonly been associated with imbalance in a single microbe, *S.aureus* [21], even though other variations are emerging, such as a reduction of *P.acnes* in AD patients [44]. *S.aureus* overcolonization of the skin surface is favoured by local AD-specific properties such as reduced skin acidity, eased bacterial adhesion and decreased antimicrobial peptide production [45]. In this context, *S.aureus* secretes a set of proteases, such as aureolysin metalloprotease, V8 and SspA serine proteases, ScpA and SspB cysteine proteases, that induce endogenous serine proteases activity in keratinocytes, leading to degradation of proteins essential for barrier integrity, such as filaggrin, kallikrein and desmoglein [23]. Thanks to its increased proteolytic activity, *S.aureus* also penetrates in the dermis, where it triggers pro-inflammatory cytokines release, thus exacerbating the disease [46]. An increase in *S.aureus* can boost AD development also via its antigen phenol-soluble modulin alpha (PSMα), which promotes production of inflammatory cytokines by keratinocytes [20]. Interestingly, PSMs by overgrown *S.epidermidis* can also induce skin inflammation and are highly present in AD both in cultured human keratinocytes and in AD mouse models [23]. This demonstrates that even commensal microbes can exert detrimental functions when the overall microbiome balance is lost. Since oxygen levels are higher in AD-inflamed skin than in healthy conditions, the whole bacteriome shifts towards an aerobic signature. This change can impair barrier function and hamper danger signaling in keratinocytes, where it is usually triggered by anaerobic cocci such as *Finegoldia*, *Anaerococcus* and *Peptoniphilus* [21]. Moreover, a correlation between microbial dysbiosis and host transcriptome changes has been described in AD, in which differentially regulated host genes in AD lesional skin samples containing *S.aureus* are functionally enriched for mainly three activities: skin barrier function, immune activation and tryptophan metabolism [21]. This finding suggests that host-microbiome interactions in skin diseases require omics integrated analyses in order to gain insights into their underlying molecular mechanisms, as we will illustrate more in detail in the following sections of this review. Finally, the host virome could also play an important role in causing AD. A recent study exploiting shotgun metagenomic sequencing of bacteriophages in human healthy skin and inflamed AD, revealed an increase of phages infecting *S.aureus* in AD-affected patients [44]. This result could be explained by increased fitness of the prokaryotic host conferred by the virus. Notably, this discovery could inform phage-based therapies to treat AD skin. This finding suggests phage-based therapies for AD, pending clarification of viral involvement and the RNA phageome’s role.

#### Psoriasis

It is a chronic inflammatory skin disease of unclear etiology, with both local and systemic symptoms, that displays hyperproliferation and altered differentiation of keratinocytes. It is characterised by genetic and autoimmune components, with hyperactivation of the immune system and an inflammatory cycle that self-sustains developing around the TNF/IL-23/IL-17 axis [47]. Psoriasis has been associated with keratinocyte production of LL-37 antimicrobial peptide able to trigger the release of pro-inflammatory cytokines and sustaining proliferation [47]. An abnormal composition of skin microbiome is observed in psoriatic skin [19] (Fig. 1C). This could contribute to immune cell hyperactivation and inflammatory responses. Indeed, antibiotic treatment in adult mice has been shown to ameliorate skin inflammation in imiquimod (IMQ) psoriatic model [48]. Several studies reported alterations in microbial diversity associated with the psoriatic plaque [49–51]. For instance, psoriasis-affected skin is associated with increased *Corynebacterium* spp. and decreased *Propionibacterium* spp. [49] along with abnormal colonization by *S. aureus* that promotes inflammation via Th17 polarization [50]. The disease also correlates with loss of *S. epidermidis* and *P. acnes*, community instability linked to *C. albicans* [19, 51] and potential alterations of new *Malassezia* species [51]. Overall, psoriatic skin displays reduced microbial diversity compared to healthy subjects [20, 52]. Interestingly, psoriatic keratinocytes can present bacterial-derived antigens to T cells, thus activating host immune response. In particular, the M protein of *Streptococcus pyogenes*, that inhabits psoriasis-associated lesions, displays molecular similarities with keratin 17. This could explain the autoimmune component of the disease, characterised by T cell activation against keratinocyte autoantigens [52]. Concerning the gut-skin microbiome axis, a real similarity between gut and skin microbes has been demonstrated for the first time in the context of psoriasis [53]. For example, *S.lentus* abundance in both psoriatic skin and faecal samples from patients differs from healthy individuals. Accordingly, oral probiotic treatments were successful in addressing psoriasis symptoms, thus confirming gut dysbiosis role in psoriasis [54].

### Skin microbiome in wound healing: friends or foes?

Wound healing is a complex, multi-step process that recruits various cell types to restore skin homeostasis, through coordinated inflammation and tissue remodeling [55]. Injured skin is vulnerable to microbial contamination; although pathogens may be present in non-infected wounds, only some establish infections that can progress locally or systemically [56]. As the epidermal barrier is re-established, also the microbiome in acute wounds seems to gradually revert to its homeostatic composition [55].

Distinct types of acute trauma, from burn wounds to surgery-related lesions, are each associated with characteristic wound microbiota compositions. Studies revealed that the most common pathogens in burns are *S.aureus*, *Escherichia coli*, *Pseudomonas aeruginosa* and coagulase-negative *Staphylococci*. Instead, common after-surgery microbes that can induce wound infection include *S.epidermidis*, *S.aureus* and *Clostridium difficile* [57].

After injury, the microbiome interacts with keratinocytes to favor or impair wound healing. For example, *S.aureus* triggers an inflammatory phenotype of keratinocytes with inflammatory cytokine release, inflammasome activation and AMP production, also causing keratinocyte death [55]. On the other hand, skin commensals, such as *S.epidermidis*, are known to promote wound healing by interacting with immune cells and keratinocytes via IL-1β and the keratinocyte-dependent IL-1 receptor (IL-1R) / myeloid differentiation primary response 88 (MyD88) signaling [55].

When inflammation persists and healing is not resolved in a short time, skin wounds become chronic. They are characterised by histopathological features such as excessive exudate, absence of granulation tissue and failure to epithelialise. Compared to acute wounds, the chronic wound microbiota generally contains more microbes than acute wounds, therefore, they often present microbial infections that can hamper proper healing [57]. A typical feature of chronic wounds is the formation of microbial biofilms [55, 58]. They consist of complex microbial communities growing in three dimensions and containing bacteria and fungi surrounded by polymeric matrices. In general, chronic wounds display reduced microbial diversity compared to healthy skin [55]. Despite the specific peculiarities of chronic wounds, bacterial genera detected in this type of wounds are similar to those found in acute injuries such as *Staphylococcus spp., Pseudomonas spp*., *Corynebacterium spp*., *Streptococcus spp*. and Gram-positive anaerobes [56]. Interestingly, anaerobic bacteria like *Enterobacter spp*. correlate with poor healing outcomes [56]. Concerning other components of microbiome, the most abundant fungal members associated to chronic wounds are *Ascomycota* and *Basidiomycota*, whereas unhealed wounds are associated with phages that infect *Enterococcus*, *Enterobacter*, *Veillonella* and *Streptococcus* species [56]. Not only microbiota composition, but also its stability over time, can contribute to the non-healing status of chronic wounds. Indeed, diabetic foot ulcers, the most studied chronic wounds, with more dynamic microbiota were shown to heal faster than those with less dynamic microbial communities [59]. In line with this, antibiotic treatments—that disrupt microbial communities’ structure - can be employed as a first-line strategy to cure infected chronic injuries.

### Skin cells-microbiome interactions in basal cell and squamous carcinoma

A seminal review by Dvorak proposed cancer as a wound that never heals [60], a concept later supported by multiple studies establishing a tight connection between chronic tissue injury and tumorigenesis [61]. Whether the skin microbiome exerts a functional role in linking these two biological processes is still unclear. Nevertheless, it has been shown that bacterial products found specifically in chronic wounds, i.e. proteins deriving from flagellates, can trigger innate immune responses via TLR5 in the skin of chronically-inflamed mouse models, thus pushing towards tumour formation [62]. Therefore, in line with the established correlation between dysbiosis and inflammatory pathways, it is not surprising that a tight association between the host microbiota and tumor initiation and progression has recently emerged in distinct cancer types [63]. In particular, bacterial species can contribute either directly or indirectly to cancer initiation and/or progression, by mechanisms ranging from the production of toxins and dietary metabolites to biofilm formation and inhibition of anti-tumoral immune responses [64]. For instance, microbial dysbiosis was reported to affect lung cancer development and progression [65]. In the context of colorectal cancer (CRC), its onset is shaped in part by the gut microbiome, which initiates inflammation and alters key signaling networks. Importantly, the emerging bacterial signatures in CRC might enable early detection as well as offer insight into likely clinical outcomes [66]. Moreover, an intriguing debate is ongoing since a number of studies identified intracellular bacteria in tumor cells where they are proposed to influence several cancer-related processes [67].

Focusing on epidermal tumors, sebaceous carcinoma represents a rare epidermal malignancy [68], in contrast to basal cell carcinoma (BCC) and squamous cell carcinoma (SCC), which are the most frequent epidermal carcinomas and constitute the bulk of global skin cancer incidence. Although no direct microbiome data are currently available for sebaceous neoplasms, the established role of the sebaceous gland in shaping skin microbial communities and the growing evidence of tumor–microbiome interactions suggest that sebaceous neoplasms are likely to harbor distinctive microbial associations.

In contrast, genome-wide association studies have already pointed out correlations between BCC and skin microbiome composition. For instance, it was reported an increase in *Staphylococcus spp*. and a decrease in *Cutibacterium spp* [69]. Another analysis further indicated the increased risk of developing BCC in association with *S.aureus*, and suggested a causal relationship between BCC and the bacterial class Gammaproteobacteria in moist skin areas [70]. Nevertheless, deeper investigation is still necessary to characterise the functional interplay between host microbiome and BCC development. Interestingly, it has been hypothesized that *Malassezia* fungi might promote BCC progression via Hedgehog (Hh) pathway [71] producing AHR ligands that can boost the growth of UV-initiated BCC. However, this still does not provide a causal relationship between skin dysbiosis and BCC. Moving towards a more systemic view, gut dysbiosis can correlate with cutaneous BCC formation in both a positive and a negative fashion [72]. For instance, bacteria belonging to the genus *Turicibacter* are thought to promote BCC via lipid metabolism, although further research is required to gain more insights into this relationship. On the other hand, gut bacteria of the genus *Ruminococcaceae* might exert a protective role against BCC growth via production of short-chain fatty acids such as pentanoate and butyrate, which in turn activate cytotoxic T cells against skin tumor cells [73]. These findings support once again the theory of a gut-skin axis, which could regulate not only skin homeostasis but also cutaneous oncogenesis.

Actinic keratosis (AK) is commonly considered a pre-tumoral lesion precursor of human cutaneous SCC. In addition to acquired genetic mutations, alterations in skin microbiota are known contributors to AK/SCC formation and progression (Fig. 1C). Microbial dysbiosis mainly consists of augmented *S.aureus*, both in relative abundance and in total load, and reduced *P.acnes*, caused by the decrease in sebum production associated with AK [70]. From a molecular perspective, *S.aureus* alters human beta-defensin 2 (hBD-2) expression by keratinocytes, thus impairing its function as tumor growth suppressor [74]. Furthermore, functional analysis of altered metagenomes revealed that in healthy skin a number of genes are associated to antioxidant activity, such as glutathione metabolism, protecting UV-exposed skin from ROS and oxidative stress; while in SCC samples, more genes correlate to *S.aureus*-specific functions. For instance, affected pathways control cell lysis and death, leading to the release of genomic DNA that can be exploited as a structural component of biofilms [75]. The role of *S.aureus* gene products in SCC tumorigenesis was also tested in vitro by exposing human keratinocytes to *S.aureus* secretome. Transcriptomic and proteomic changes revealed a downregulation of cell cycle and DNA repair, and induction of oxidative stress markers [76]. In light of recent findings on bacterial dysbiosis in SCC, new treatment strategies target skin flora. For example, abrogation of the microbiome by antibiotic treatment reduced the tumour burden, suggesting a functional role of the microbiome in SCC therapy response [77]. Finally, besides the detrimental role of microbial imbalance in SCC, it has been hypothesised that *Malassezia spp*. could represent a protective component of the skin microbial community, since it was detected mainly in perilesional healthy skin [78].

Although multiple data support a functional role of distinct cutaneous microbes in modulating epidermal tumor formation, the involvement of the skin microbiome in tumorigenesis remains relatively underexplored despite increasing research efforts. Indeed, data addressing the full spectrum of cross-kingdom interactions remain very limited in the field of skin oncology. Therefore, a deeper understanding of the molecular and biochemical crosstalk underlying skin tumorigenesis will be important to identify potential new targets for cancer therapy.

### Therapeutic approaches based on host-microbe functional crosstalk

Several therapeutic strategies based on microbiome modulation are currently employed, in particular on inflammatory diseases as their host-microbiome association is more understood [56]. Antibiotic treatment has been the frontline choice for medication of skin wounds and other cutaneous disorders for a long time, including both oral and topical administration. However, its lack of selectivity and the inability to reach deeper regions of microbial biofilms make this strategy less efficacious. Moreover, antimicrobial resistance has emerged as an increasing threat to human health. Strikingly, novel therapies leveraging a target-specific approach are being developed and seem to represent effective treatments also in severe chronic wound infections [79]. For instance, phage therapy consists in delivering bacteriophages to the patient. These viral entities that selectively infect and kill bacteria have shown success in fighting *Staphylococcus aureus* and *Pseudomonas aeruginosa* skin infections [80]. Another promising approach is represented by the use of probiotics, i.e. live microorganisms with beneficial effects on the recipient individual, as in the case of probiotic preparations derived from *Lactobacillus spp*. that reduce bioburden in burn wounds [81]. A novel therapeutic option is genetic and/or metabolic engineering of live bacteria. For instance, engineered *C.acnes* strains have been successfully tested in vivo as a platform to modulate sebum production in acne models [82]. Finally, microbial transplantation represents a valuable tool to ameliorate skin dysbiosis through selected skin commensals as well as diverse microbial consortia. Of note, since the gut-skin microbiome axis can contribute to cutaneous homeostasis and pathogenesis, the use of fecal microbiota transplantation represents a potential innovative strategy to cure skin inflammatory conditions [83]. For instance, AD-related inflammatory responses could be attenuated after fecal transplantation in mouse models of AD, by modulating the release of inflammatory cytokines [84].

As illustrated, current microbiome therapies mainly focus on bacteria, but the skin virome and mycobiome are likely to emerge as targets for future dermatological treatments due to their roles in skin homeostasis. However, deeper functional characterization of skin commensals, including the identification of interindividual variations and their interactions, is needed. Integrating meta-omics with host transcriptomics in skin microbiome research offers opportunities even for personalized dermatology. Linking microbial functions to host gene expression can uncover biomarkers and therapeutic targets, while machine learning and computational biology enable analysis of complex datasets, prediction of interaction networks, and discovery of key microbial drivers. Such multi-omics approaches will be central to understanding how to restore skin homeostasis and to translating basic research into clinical applications. Nevertheless, translating computationally predicted therapies into practice remains limited by difficulties in establishing causality, highlighting the need for more robust datasets linking dysbiosis to cutaneous pathogenesis and for direct functional validation.

## Omics technologies and analytical tools

### Studying the microbiome composition

Omic-based microbiome studies provide insights that fall into two main, often separated categories: community composition and interaction with host cells. In terms of composition, research focuses on comparing microbial community composition across healthy and diseased skin sites or between different anatomical regions. Traditional culture-based methods to study the cutaneous microbial landscape miss many host-adapted microbes, whereas culture-independent approaches like sequencing and metagenomics reveal a broader, more complete view of microbial communities directly from samples [85]. The most common approach involves the targeted amplification of molecular fingerprints specific to certain microbial groups. For bacterial communities, the 16S ribosomal RNA (rRNA) gene is targeted, as it is highly conserved across all bacteria, with variable regions allowing for species differentiation [86]. In the case of fungi, the internal transcribed spacer 1 (ITS1), a non-coding DNA region located in the rRNA gene cluster is used [87]. After amplification, nucleic acid sequencing of these regions is performed to accurately identify species or even strains. Unlike bacteria or fungi, viruses lack universal markers, so shotgun metagenomic sequencing is used. This approach sequences all genetic material present in a sample, enabling the detection of both known and novel viruses. Despite their advantages, sequencing methods have limitations, including amplification biases, challenges in species resolution and difficulty in understanding functional roles [88]. In particular, despite being less expensive and robustly validated, the 16S amplicon sequencing cannot always resolve microbes to the species or strain level. On the contrary, shotgun metagenomics has improved taxonomic resolution power, but requires higher costs and its sensitivity is still not optimised for low abundant strains in mixed communities.

**Fig. 2:**
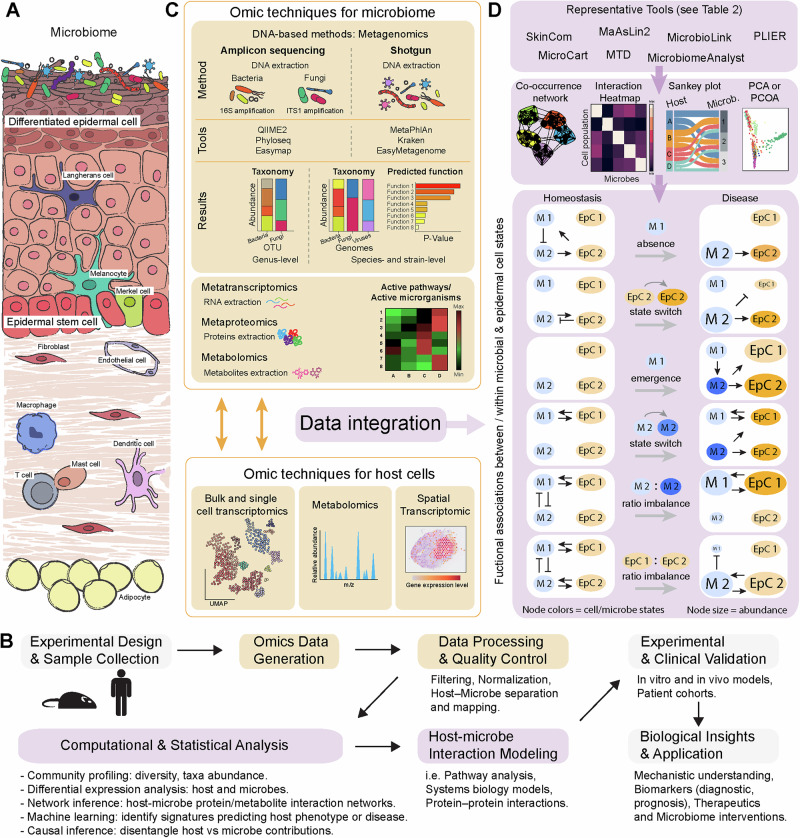
Omic approaches to characterise the host microbiome composition and interactions in the skin. **A** Diagram of the skin cellular components, showing epidermal and dermal layers, and associated surface microbiota. **B** Schematic workflow illustrating how omics can be integrated through computational methods to study host–microbe interactions. These steps include the acquisition of omics from host cells and microbes to map their composition and individual features (light orange rounded rectangles, later expanded with details in (**C**)), the data integration to decipher the host-microbiome functional interactions (pink rounded rectangles, later expanded with details in (**D**)). **C** Overview of omic techniques applied to both microbes (e.g., amplicon sequencing and shotgun metagenomics, metatranscriptomics, metaproteomics and metabolomics) and host cells (e.g., bulk and single-cell RNA-seq, metabolomics and spatial transcriptomics), enabling taxonomic, functional, and activity-based profiling. **D** Integration of omic data using computational tools supports network analysis, dimensionality reduction, and visualization of host–microbe associations. Conceptual models illustrate dynamic interactions between microbial taxa (M1, M2) and epidermal cell states (EpC1, EpC2) across homeostasis and disease, including transitions, imbalances, and state switches. Node size and color indicate relative abundance and identity, respectively.

To decipher microbiome composition from these data, several bioinformatics tools have been developed, each focusing on specific aspects of microbial community profiling. Table 1 provides a comparative overview of these tools, detailing their primary functions, applications, output and distinctive features to analyse shotgun metagenomics and 16S rRNA sequencing [89–98]. For example, MetaPhlAn [89] and Kraken [90] enable taxonomic classification from shotgun metagenomic data, while QIIME2 [91] (Quantitative Insights into Microbial Ecology), RDP (Ribosomal Database Project) Classifier [98] and Phyloseq [92] provide pipelines for analyzing 16S rRNA sequencing data. Databases such as SILVA [93] and Greengenes2 [94] support taxonomic assignment, while MGX [95], EasyMap [96] and EasyMetagenome [97] offer user-friendly tools for quality control analysis, data visualization and functional analysis (Fig. 2B, C).

**Table 1:** Computational tools to assess microbiome composition.

Sequencing Methodology	Tool	Primary function	Biological question	Microbiome Input Data	Distinctive Features	Output	References
Shotgun	MetaPhlAn	Species-level taxonomic profiling using clade-specific markers.	*What species are present and how do they vary across conditions?*	Shotgun metagenomics	High-resolution and robust species-level profiles; widely adopted.	Relative abundance tables, species heatmaps.	Blanco-Míguez et al., [89]
	Kraken	Ultra-rapid taxonomic classification of metagenomic reads.	*Which microbes are present in large datasets?*	Shotgun metagenomics	Extremely fast and scalable; suited for high-throughput datasets.	Classification reports, abundance tables.	Lu et al., [90]
	EasyMetagenome	Pipeline for multiple analysis methods, including quality control (QC), host read removal, assembly.	*How can I perform a complete metagenome workflow of shotgun data with minimal coding?*	Shotgun metagenomics	User-friendly pipeline; integrates multiple analytical steps.	QC reports, contig/bin assemblies, abundance tables.	Bai et al., [97]
	QIIME2	Provides complete and integrated workflow from raw sequencing reads to publication-quality visualizations and statistics analysis, focusing on diversity and taxonomy.	*How do microbial communities differ across different conditions?*	16S rRNA, shotgun	Community-driven and extensible; ensures reproducibility through provenance tracking and standardized outputs; ideal for comparative microbiome studies.	OTU/ASV tables, alpha/beta diversity plots, taxonomic summaries.	Bolyen et al., [91]
16S	Phyloseq	R package for statistical analysis and visualization.	*How can I statistically compare microbial communities?*	16S rRNA, shotgun	Powerful visualization and statistical integration in R.	Rich graphics, ordination plots, diversity statistics.	McMurdie & Holmes, [92]
	MGX (Microbiome Genomics)	Workflow for 16S, shotgun metagenomics and amplicon sequencing data. It integrates taxonomic profiling, functional annotation, and statistical analysis.	*How do taxonomy and function change in the microbiome?*	16S rRNA, shotgun	Flexible platform that combines taxonomic and functional metagenome analysis within one environment. It offers ready-to-use workflows, interactive visualizations, and the possibility to add custom pipelines or data.	Combined taxonomic + functional profiles.	Jaenicke et al., [95]
	Greengenes2	A 16S rRNA database that offers tools for taxonomic classification and microbial profiling.	*Which microbes can be reliably classified in 16S datasets?*	16S rRNA	Updated and curated; improves classification accuracy.	Taxonomic reference tree, annotated taxa.	McDonald et al., [94]
	RDP (Ribosomal Database Project) Classifier	Naive Bayes–based classifier for taxonomic and phylogenetics assignment of rRNA sequences.	*Which taxa are present in my 16S data?*	16S rRNA	One of the earliest and most widely validated classifiers for microbial taxonomy; supports local use, and the RDP Classifier (v2.14, 2023) remains actively maintained and integrated into major microbiome pipelines.	Taxonomic labels with bootstrap confidence scores.	Cole et al., [98]; RDP Classifier v2.14, 2023
	SILVA Database	Curated, high-quality reference database of small and large subunit rRNA sequences for taxonomic assignment and phylogenetic analysis.	*Which taxa are present in my 16S rRNA data?*	16S rRNA	Provides one of the most comprehensive rRNA sequence collections across all domains of life. Continuously updated and curated. Offers QC alignments and standardized taxonomy. Considered a gold-standard reference in microbial ecology studies.	Reference taxonomy, annotated alignments.	Quast et al., [93]
	EasyMap	Web tool for 16S taxonomic assignment + functional prediction.	*How can I perform a complete metagenome workflow of 16S data with minimal coding?*	16S rRNA	User-friendly and interactive platform; Offers complete microbiome analysis (QC, taxonomy assignment, microbial composition, alpha and beta diversity, differential abundance analysis and functional prediction.	Taxonomic tables, diversity plots, functional predictions.	Dahan et al., [96]

### Omics integration and single cell omics for deep insights

By integrating omics data, researchers are exploring microbiome-host molecular interactions and composition differences in pathological contexts affecting epithelial barrier tissues. For instance, by combining the transcriptomic analysis RNA-seq of host bronchial epithelial cells and 16S rRNA gene sequencing, an oral microbial signature in lung cancer patients was linked to phosphatidylinositol 3-kinase (PI3K) signaling activation [99]. Specifically, a positive correlation was reported between oral commensal bacteria such as *Megasphaera* and *Veillonella*, identified through QIIME (Table 1), and lung cancer-related signaling pathways, including ERK/MAPK and PI3K/AKT [99]. In another study, RNA-seq data of intestinal biopsies from ulcerative colitis patients were analysed with QIIME, revealing an inverse correlation with the abundance of *Akkermansia* and *Bifidobacteria*, microbes known for their anti-inflammatory properties [100]. While these studies illustrate the potential of omics to connect microbiome dynamics with host health in other epithelia, gaps remain in understanding skin microbiome–cell interactions. Several powerful tools like CellChat [101] have been developed to analyze cell-cell communication between keratinocytes and other cell types in skin, but their application to the crosstalk with the microbiome still needs to be explored. However, combining transcriptomics data from RNA-seq with paired metagenomic analysis allows the identification of specific host pathways that are activated or suppressed in response to microbial interactions.

For instance, shifts in the microbial community, driven by environmental changes [102], skin disorders [21, 103], or therapeutic interventions [104, 105] have been correlated with alterations in keratinocyte gene expression, affecting processes such as differentiation, metabolism, cytokine production, and the release of metabolites and antimicrobial peptides that defend against pathogens.

This integrated approach is particularly valuable in studying skin conditions where microbial dysbiosis and changes in keratinocyte function are strongly linked, such as AD. In this context, for example, *S.aureus* dysbiosis was shown to induce increased proteolytic activity in human keratinocytes at mRNA and protein levels [46], resulting in a disruption of the skin barrier and abnormal host immune responses [106]. In colorectal cancer studies, similar multi-omics approaches have revealed correlations between host metabolites and microbial taxa. Specifically, metabolites like 5-aminovalerate were associated with *Adlercreutzia*, while cholesteryl ester correlated with *Staphylococcus*, *Blautia*, and *Roseburia*, relationships linked to tumorigenesis in colorectal adenomas [107]. Moreover, integrating host transcriptomic data with gut microbiome profiles obtained by QIIME has uncovered genes and pathways related to inflammation, gut barrier protection, and energy metabolism [108]. Although focused on gut microbiome, these studies highlight that RNA-seq data can provide insight into the expression of key inflammatory mediators, such as cytokines and chemokines, by epithelial cells, helping to establish direct links between microbial dysbiosis and inflammatory pathways.

Recent advances in spatial omics have also provided new insights into the organization of microbial communities within the skin and their interactions with host cells. For example, spatial transcriptomics and metagenomics revealed how spaceflight-induced stressors impact both the skin microbiome and host inflammatory responses [109]. Frameworks such as MicrobioLink [110] (Table 2), which integrates host and microbial molecular interaction networks, and MicroCart [111] (Table 2), which supports spatial and temporal multi-omics data integration, further expand the ability to capture host–microbiome crosstalk. Omics integration has also enabled the identification of location-specific microbes, such as the distinct microbial signatures associated with different regions of the HF microbiota [112]. Furthermore, spatial diversity of the skin bacteriome has been characterised across different body sites, highlighting distinct microbial communities that correlate with physiological and pathological states [113]. Moreover, longitudinal studies examining temporal and spatial variation in the skin microbiome have demonstrated differences in microbial composition between healthy individuals and patients with AD [114].

**Table 2:** Omics integration to dissect host-microbe functional interactome.

Goal	Tool	Primary function	Biological question	Microbiome Data	Host Data	Distinctive Features	Output	References
Data Integration & Association	MicrobioLink	Study microbe–host protein interactions in host molecular networks.	*How are host pathways modulated by microbial proteins?*	Metaproteomics	Transcriptomics	Framework mapping microbial–host protein interactions using network diffusion.	Network diagrams, interaction tables.	Gul et al., 2025
	MicroCart	Study Microbiome-Host interaction by spatial multi-omics integration in tissue context.	*How are microbes and host signals spatially organized?*	16S rRNA	Spatial omics	Enabling spatial multi-omics integration.	Spatial maps, cell–microbe overlays.	Zhu et al., 2025
	MTD	Joint analysis of microbial and host transcriptomes (bulk & single-cell)	*How do host and microbial transcriptomes co-vary?*	Metatranscriptomics	Bulk & scRNA-seq	User-friendly tool supporting bulk+single-cell host–microbe transcriptomics within a single workflow of analyses.	Expression matrices, co-variation networks, Heatmap of Differential Expressed Genes (DEG) and Functional Pathway Enrichment.	Wu et al., 2022
	MaAsLin2	Multivariate statistical framework linking microbial features with host molecular and clinical data.	*Which microbial features correlate with clinical traits?*	Multi-omics (metagenomics, metabolomics, transcriptomics)	Transcriptomics, Proteomics, Phenotypes	Widely used command line or R tool for high-dimensional association testing with covariates.	Regression tables, feature–trait association plots.	Mallick et al., 2021
	PLIER	Uses matrix factorization to extract shared pathways across host-microbiome multi-omics datasets.	*Which pathways are shared in host and microbiome datasets?*	Multi-omics (e.g., metagenomics, metabolomics)	Transcriptomics, Proteomics, Other omics	Identifies pathways that regulate gene expression across heterogeneous datasets.	Pathway activity scores tables and Heatmap.	Mao et al., 2019
	mixOmics	R package dedicated to the multivariate analysis of biological datasets and multi-omics integration.	*How do microbial features integrate with host omics?*	Metagenomics, metabolomics	Transcriptomics, metabolomics	R package; Provides a wide range of methods for data integration, biomarker discovery, and data visualisation.	Correlation networks, Correlation Heatmap and Circle plot.	Rohart et al., 2017
	HONMF	Tool for Cross-kingdom microbiome integration.	*Which cross-kingdom and host–microbiome associations can be uncovered?*	Metagenomics (bacteria, fungi, viruses), metatranscriptomics	Transcriptomics, proteomics	Identifies kingdom-specific latent factors; integrates them across kingdoms; captures higher-order microbial interactions.	Clusters, cross-omics networks.	Ma et al., 2023
Functional & Metabolic Inference	MetScape	Cytoscape plugin for host metabolic networks.	*How do host metabolic pathways link to microbial metabolism?*	Metabolomics, functional metagenomics	Transcriptomics, metabolomics	Network-level visualization of metabolic host–microbe interactions.	Network graphs, pathway diagrams.	Basu et al., 2017
	MicrobiomeAnalyst2.0	Web-based analysis of microbiome, metabolite, and phenotype data.	*How are microbial profiles aligned with metabolites/phenotypes?*	Metagenomics, metabolomics	Clinical phenotypes	User-friendly web-based tool. Complete Raw Data Processing; associations between community compositions and metabolic activities; integration of datasets across multiple studies.	Differential analysis and interactive visualizations for popular graphical outputs, statistical reports.	Lu et al., 2023
	MIMOSA2	Infers microbial metabolic differences in metabolite levels across sample to identify taxonomic contributors to metabolite variation.	*Which microbes drive observed metabolite changes?*	Metagenomics, metabolomics	None	Focused on linking taxa to metabolite changes with contribution scores.	Contribution tables, predicted metabolite profiles.	Noecker et al., 2022
	PICRUSt2	Predicts function profiles from 16S rRNA data using KEGG pathways.	*What functions can be inferred from taxonomy using 16S data?*	16S rRNA	None	Extends 16S surveys into predictive functional inference without shotgun data; provides KO/MetaCyc tables comparable with common shotgun metagenomics outputs.	KO/MetaCyc tables, functional heatmaps.	Douglas et al., 2020
	HUMAnN	Part of bioBakery3 meta-omic tool suite, allows to profile metabolic pathways from shotgun metagenomes.	*What metabolic functions are encoded by communities?*	Metagenomics, metatranscriptomics	None	High-resolution, taxon-stratified microbial pathway profiling.	Pathway abundance tables, Heatmap Functional profiles/samples.	Franzosa et al., 2018
Predictive & Simulation	MICOM	Python package to study microbial communities using metabolic modeling.	*How do microbes alter host metabolism under different conditions?*	Metagenomics, metabolomics	Simulated host	Mathematical modeling framework that can recapitulate the growth rates of diverse bacterial species to simulate metabolic interactions within microbial communities.	Flux balance models, predicted metabolite exchanges.	Diener et al., 2020
	MAGMA (rMAGMA)	R package for detecting interactions between microbiota that takes into account the noisy structure of the metagenomic count data.	*Which microbial taxa are ecologically associated within the community?*	Metagenomics	None	R package; corrects for compositional bias, sparsity, and noise in microbiome data to infer reliable association networks.	Sparse interaction networks (graphs).	Cougoul et al., 2019
	SkinCom	Tool for investigating microbe-metabolite interactions; it facilitates experimental in vivo design.	*What are the effects of external exposures on skin microbial community structure and function?*	16S or shotgun	Environmental data	Synthetic modeling framework designed to assess environmental and chemical exposure effects on microbial communities.	Simulated skin community models, exposure outcomes.	Lekbua et al., 2024
	SkinBug	AI-based predictions of microbial metabolism and effects on host tissue due to exogenous inputs.	*How are compounds metabolized by the skin microbiome?*	Metagenomics (AI-based)	Exogenous molecule data	AI-driven predictions of microbial metabolism; Focus on xenobiotics, cosmetics, and drugs effetcts.	Predicted metabolic pathways, host impact predictions.	Jaiswal et al., 2021
	MMVEC	Neural network that predicts metabolite abundance profile from microbe sequence through microbiome–metabolome integration.	*Which microbes are associated with specific metabolites and viceversa?*	Metagenomics, metabolomics	Metabolome	Integrates microbiome and metabolome; interactive visualization. Predicts metabolites abundance profile from microbe composition.	Heatmap, Feature plot, Network, predictive scores.	Morton et al., 2019
	PM-CNN	Organizes microbes based on their phylogenetic relationships and extracts features using a multi-path convolutional neural network.	*Can microbial pathway data predict host phenotype?*	Metagenomics	None	Machine learning based prediction model; Incorporates phylogenetic relationships; multi-path analysis; improved classification accuracy.	Classification, Phylogeny tree, Prediction scores.	Wang et al., 2024
	MicroKPNN-MT	Predicts human phenotypes based on microbiome data and additional metadata.	*How do microbes contribute to multiple host traits?*	Metagenomics	Phenotype/clinical data	Machine learning model with high prediction accuracy and interpretability; samples additional metadata could be used as input features for improved prediction.	Prediction scores.	Monshizadeh et al., 2025

By integrating omics approaches, researchers are beginning to unravel the intricate molecular dialogue between the skin microbiome and its host, moving beyond correlations towards mechanistic insights. These analyses could be further enhanced by the resolution of recent and upcoming technologies that allow to perform single cell omics of epithelial cells but also bacteria [115–117].

The convergence of transcriptomics, including expression profile analysis at single cell resolution, metagenomics, and spatial omics not only is enhancing our understanding of skin health and disease but is also opening paths for precision diagnostics and targeted therapies.

### Host-microbe interactions and network analysis

As previously discussed, host–microbe interactions are crucial for maintaining skin immunity, barrier function, and overall homeostasis, and defending against pathogens. Decoding and predicting these interactions is possible through a computational intersection of data from omics within functional networks. Therefore, metagenomic sequencing, in combination with metatranscriptomic, metabolomic, and metaproteomic approaches, enhances the functional interpretation of microbial communities and their interactions through network-based analysis that enables visualization and interpretation of how microbes interact with each other and with host cells (Fig. 2B,D; Table 2). By constructing correlation networks, interaction patterns can be uncovered to investigate structural changes in microbial communities in response to disruptions, such as environmental stress or disease [118, 119]. These networks provide a map of relationships within microbial ecosystems, capturing both direct interactions (such as physical contacts or metabolite exchange between microbes and host cells) and indirect interactions (mediated by environmental changes or other community members). Additionally, they can incorporate metabolites produced by microbes or clinical traits linked to host health, creating a more comprehensive understanding of the system complexity and microbiome balance. Understanding the changes from skin health to dysbiosis via network analysis can help identify new targets for therapies by detection of microbial and molecular biomarkers. For example, co-occurrence networks of microbiome sequencing and metabolomics data from healthy adult subjects and AD patients were calculated with MMVEC [120] (Table 2), a QIIME2 plugin for estimating microbe-metabolite interactions. [121] This revealed higher co-occurrence of certain metabolites and bacteria in healthy individuals and non-lesional skin compared to AD-lesional skin [121].

### Microbiome cross-kingdom interactions and system-level insights

As the skin is a dynamic environment where bacteria, fungi, and viruses coexist and interact, investigating cross-kingdom relationships and their collective effects on host cells is a complex but promising direction in skin microbiome research. Through integrated metagenomic analyses, researchers can explore how different microbial kingdoms cooperate or compete, and how these interactions shape the skin immune system and keratinocyte behavior. For example, fungal species such as *Malassezia spp*. are known to coexist with bacteria on the skin, and changes in their abundance may affect keratinocyte responses differently from bacterial shifts alone [122]. In particular, *Malassezia spp*. can produce enzymes that limit *S.aureus* virulence and biofilm formation, suggesting a protective role in maintaining skin health [123]. As mentioned above, the virome strongly influences the microbiome, as certain viruses—such as mycoviruses infecting *Malassezia spp*.—can alter fungal physiology and host interactions, thereby affecting pathogenicity and skin health [32, 124].

Although most of these studies rely on descriptive or correlation-based analyses, integrative bioinformatic frameworks hold great potential for advancing the field. Tools such as QIIME2, MetaPhlAn, and HUMAnN [125] (Tables 1 and 2) enable reproducible and high-resolution functional profiling of microbial communities, while multivariate integration platforms like MixOmics [126] (Table2) and MTD (Meta-Transcriptome Detector) [127] (Table 2) or matrix factorization approaches such as PLIER [128] (Table 2) and HONMF [129] (Table 2) have been developed to integrate heterogeneous omics data and uncover cross-kingdom associations. For example, the HONMF framework has been applied to microbiome multi-omics datasets to jointly analyze bacterial, fungal, and viral profiles, enabling clustering, feature selection, and the discovery of interaction patterns across kingdoms. In the context of skin, combining metagenomics and metabolomics allowed the identification of three distinct metabolite–microbe clusters, linking specific bacterial taxa such as *Cutibacterium* and *Staphylococcus* to metabolite signatures associated with skin barrier and metabolic features [130]. Applying such approaches to skin microbiome research could help move from pairwise observations toward a true systems-level understanding of how bacteria, fungi, and viruses collectively influence keratinocyte biology, immune responses, and barrier functions.

### Artificial Intelligence and machine learning tools to unravel microbiome-host molecular and metabolic interactions

Artificial intelligence (AI) represents a recent powerful tool in advancing our understanding of the intricate interplay between and within epithelial cells and the skin microbiome. Machine learning algorithms, such as those implemented in QIIME [91], rapidly analyze large-scale metagenomic data, identifying shifts in microbial communities that impact epithelial barrier functions and immune responses. While QIIME primarily relies on statistical and sequence alignment methods, it also integrates machine learning approaches, such as Naive Bayes classifiers [131], for taxonomic assignment. These features help map out the dynamics of microbial diversity on the skin surface, crucial for studying conditions like eczema or acne where the microbiome-epithelial balance is often disrupted.

These methods, although simple, remain widely used because they are reproducible and easy to interpret, qualities that are sometimes missing in more complex AI models.

In contrast, through machine learning and deep learning, AI allows the development of predictive modeling tools targeted to skincare and therapeutic interventions. TensorFlow [132] and PyTorch [133], two open-source deep learning frameworks, have been applied to build models predicting associations between microbial compositions and epithelial health [134, 135]. PM-CNN [136] (Phylogenetic Multi-path Convolutional Neural Network) is a model that enables the classification of multiple microbiome states and the detection of disease signatures using microbial composition data. Another powerful tool, MicroKPNN-MT [135], predicts human phenotypes based on microbiome data and additional metadata, such as age and gender, facilitating multi-disease prediction.

Deep learning approaches are also increasingly applied to histological and imaging data. Tools like CellProfiler [137] and DeepCell [138] enable high-throughput image analysis of epithelial tissues. While DeepCell leverages neural networks to distinguish cellular features and model spatial organization, CellProfiler focuses on feature extraction and can be combined with machine learning models for downstream analysis. These platforms can quantify changes in cellular morphology and epithelial responses to microbial interactions, providing insights into how microbial populations might influence epithelial integrity and inflammation over time. The complexity of these models can come at the cost of transparency and reproducibility, particularly in small or heterogeneous cohorts.

Explainable AI (XAI) [139] represents an emerging solution to the need for transparency and user confidence in decision-making processes, while also supporting reproducibility by highlighting the microbial and host features that consistently drive model predictions across cohorts. An XAI model was used to predict phenotypes like skin hydration, age, menopausal status, and smoking status based on previously available skin microbiome data, identifying microbial signatures linked to these traits and explaining their relationships with phenotypic variations [140].

As AI tools continue to evolve, their capacity to integrate omics data combining genomics, transcriptomics, and metabolomics will be important for a holistic understanding of the skin complex ecosystem. By revealing how microbial interactions shape epithelial cell responses at a molecular level, AI-driven research holds transformative potential for advancing dermatology and microbiome-related therapies.

Advancements in AI have catalyzed new methods for studying the interaction between the skin microbiome and epithelial cells, enabling researchers to decode the complex symbiotic and pathogenic relationships at the cellular and molecular levels, including metabolic insights (Fig. 2). Tools like MIMOSA [141] (Microbial Metabolic Interactions in the Microbiome), MicrobiomeAnalyst2.0 [142] and PICRUSt [143] (Phylogenetic Investigation of Communities by Reconstruction of Unobserved States) are widely used in microbiome research to predict the functional potential of microbial communities and their impact on the host (Table 2). For instance, MIMOSA applies machine learning to metabolic network models to identify how specific microbial metabolites interact with host cells, potentially affecting epithelial barrier integrity or immune responses. For the analysis of host-microbiome gene expression interactions, platforms like MaAsLin [144] (Multivariate Association with Linear Models) offer a data-driven approach to link microbial community composition with host transcriptomic data (Table 2). iHMP Data Portal [145] (Integrative Human Microbiome Project) provides access to a wide range of multi-omics datasets, which can be used to study microbiome-host interactions across various body sites, including the skin. Meanwhile, HUMAnN [125] (HMP Unified Metabolic Analysis Network) is used to reconstruct host-microbiome metabolic interactions at high resolution, linking microbial pathways with host metabolic responses (Table 2). MICOM [146] (Microbial Community Modeling) is a computational tool for modeling microbial communities and predicting their metabolic interactions with the host (Table 2). Metabolic modeling tools like MAGMA [147] (Microbiome analysis with Global Metabolic Models) combine metabolic modeling with omics data to predict how microbial metabolism impacts the host (Table 2). Cytoscape, a widely used bioinformatics software for visualizing molecular interaction networks, can be enhanced with plugins like MetScape [148] to explore microbiome-host interactions at a network level (Table 2).

Looking ahead, such models could drive personalized skincare and treatments by tailoring interventions to individual microbiome profiles. They may also enable non-invasive health monitoring, using skin microbiome data to detect broader health indicators such as immune responses or metabolic conditions and support predictive dermatology by anticipating skin issues and allowing for customized preventative care. Additionally, skin-specific platforms such as SkinCom [149] (Table 2) and SkinBug [150] (Table 2) illustrate how computational models can simulate microbial–host interactions and predict responses to external exposures, offering opportunities for pharmaceutical and cosmetic industries to develop microbiome-targeted products informed by bacterial signatures tied to skin health and aging. Future innovations might even include real-time feedback systems, like wearables or smart patches, that track microbiome shifts and provide instant insights into how lifestyle factors affect skin health.

In summary, these tools should be applied selectively, guided by the research question. While QIIME-like frameworks are best for reproducible and interpretable surveys, deep learning models provide strong predictive power but require careful validation, and metabolic modeling uniquely links microbial alterations to host functional pathways. The choice of tool should therefore reflect the biological question: reproducibility for clinical contexts, prediction for hypothesis generation, or mechanistic depth for systems biology.

## Microbiome and epithelial memories

**Fig. 3:**
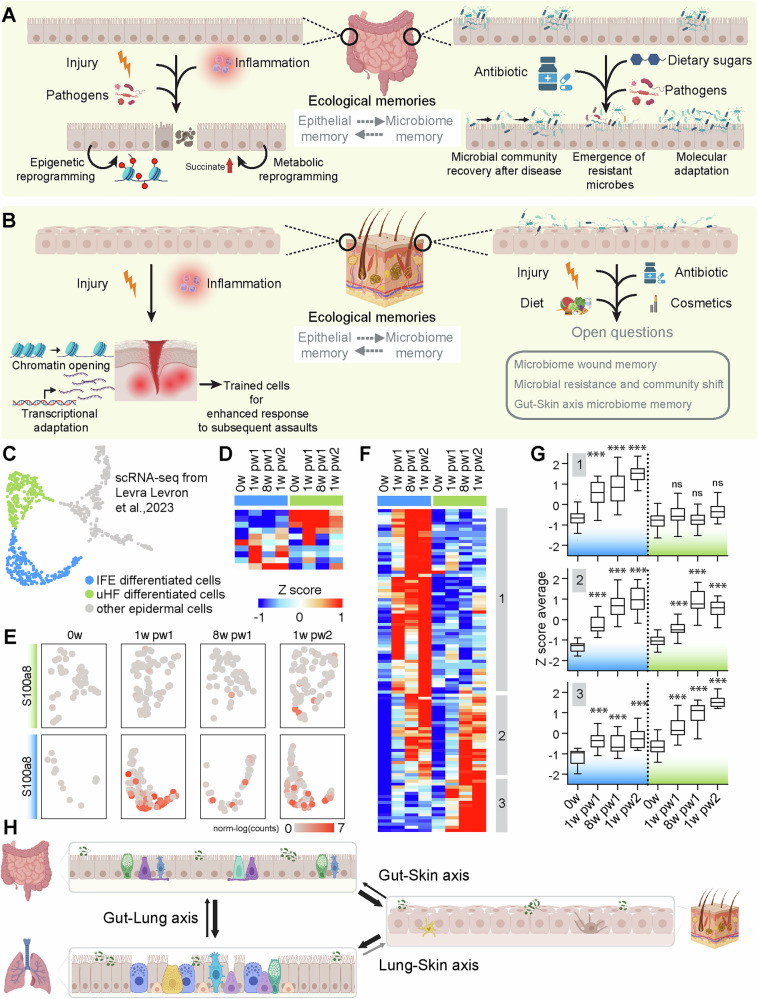
Ecological memories in barrier epithelia. Epithelial and microbial memories in the gut (**A**) and in the skin (**B**). A potential reciprocal crosstalk is hypothesised between epithelial cells and tissue-resident microbes. **C** UMAP visualization of scRNA-seq data [161] highlighting differentiated interfollicular epidermal cells (IFE) in blue and differentiated upper hair follicle cells (uHF) in green. **D** Heatmap of AMP genes across different time points and distinct epidermal differentiated cells regions (IFE and uHF) at baseline (0w), 1 week post first wound (1w_pw1), 8 weeks post first wound (8w_pw1, established memory), and 1 week post second wound (1w_pw2). **E** Gene expression maps projected onto UMAP embeddings, showing expression dynamics of a representative AMP gene in uHF (green) and IFE (blue) differentiated compartments across time. **F** Heatmap of metabolic genes across different time points and epidermal differentiated cells in distinct areas (IFE and uHF). **G** Z-score average of the three gene clusters highlighted in grey in (**F**). **H** The gut-lung-skin axis is reported to connect three epithelial barrier tissues. Interactions are known to be bidirectional, but evidence is still lacking for some of the links, particularly to cellular memories. The draft of (**A**, **B**, **H**) were created with BioRender.com.

The concept of microbe ecological memory has been proposed by recent studies showing that microorganisms inhabiting the gut can remember previous exposure to dietary compounds, thus reshaping their community structure and function [151]. Specifically, metabolic enhancement was observed soon after exposure to the carbohydrate inulin in a restricted number of intestinal bacteria, followed by a more widespread transcriptional adaptation of neighboring microbes. This phenomenon is defined as an ecological memory, given that past events affecting the gut microbial ecosystem can impact on its future response to environmental stimuli. In addition to dietary oscillations, host microbes can also undergo community adaptation after antibiotic treatment, as shown in pneumococcal disease [152], or after infections by pathogens causing acute diarrhea [153]. These adaptive behaviors improve the response to subsequent perturbations in a similar way as for the immune system. In line with these discoveries, intestinal stem cells were shown to retain epigenetic marks of their past encounters with harmful stimuli, such as bacterial infections [154, 155] and inflammation [156]. This long-lasting memory affects the ability of gut epithelial cells to adapt to subsequent challenges, in both beneficial and detrimental ways (Fig. 3A). Interestingly, epigenetic memories of an assault can also be stored in multiple epidermal cells [157, 158] and shared by damaged and tumor-forming cells across several epithelia, including the skin [159, 160]. Indeed, previous work revealed that epithelial stem cells of the skin are able to acquire wound memory also distally from the lesion site, that predisposes to skin cancer [161]. However, the mechanism by which cells located far from the wound sense tissue damage and establish a persisting memory is still unknown. Therefore, the intriguing question arises whether the microbiome and epidermal cells carry a reciprocal memory of previous injurious events. Further investigation is needed to reveal whether skin microbes contribute to this process via altered community composition and dysregulated interactions with surrounding epidermal cells (Fig. 3B). We also hypothesize that by harnessing these putative skin ecological memories, new therapeutic approaches could be designed in order to improve tissue repair potentially also in chronic wounds.

A further layer of complexity is represented by the heterogeneity of host-microbe molecular and metabolic responses in distinct epithelial niches (Fig. 3C-G). Indeed, previous work highlighted that epithelial wound memory is lineage-specific, with a well-defined HF stem cell population undergoing a peculiar adaptation after injury [161]. Additionally, AMP and metabolic gene expression by keratinocytes differs depending on the skin compartment [161–163] (Fig. 3D-G). Consequently, it is difficult to avoid hypothesising the existence of niche-specific host-microbe interactions that are functional for tissue memories in epithelial barriers such as skin. In addition, as a gut-lung-skin axis is reported [164, 165], there might be an inter-tissue crosstalk influencing cellular and microbial memories (Fig. 3H).

## Challenges and outlook

A comprehensive analysis of the host-microbiome molecular and biochemical interactions is essential to advance our understanding of the mechanisms underlying barrier epithelia homeostasis and skin disease. While the gut environment has been more widely explored, research in the field of skin biology only started to unveil a limited fraction of the whole host-microbe interactome and their functional interplay. State-of-art studies now provide insights into the crosstalk involving specific microorganisms in selected pathological conditions, but fail in framing the bigger picture. Nonetheless, an ever-growing toolbox of omics and computational methods is now available to expand our knowledge. Therefore, a more widespread use of these approaches is required to reveal fundamental aspects of epithelial barriers biology. We anticipate that the combination of these tools to integrate omic data will allow the identification of new potential targets to develop future therapies, even though inferring causality will be challenging due to the current gap between in silico predictions, in vivo spurious co-occurrence correlations and cause-effect relationships. Also, the taxonomic resolution of microbiome datasets in the cutaneous ecosystem is currently limited, thus suggesting a crucial need for improvement of current sequencing methods to gain insights at strain level. Indeed, more precise identification of pathogenic and beneficial strains is emerging as fundamental to bridge the existing gap between predictions and clinical applications. Together with exhaustive experimental validation, strain-level detection and culturing will finally allow the development of biotechnological drugs able to reach dermatological use. Yet, examples of successful treatments based on the host-microbe interaction studies already exist, as illustrated in this review [56, 79–84]. Concerning the use of AI to identify new diagnostic markers and targets for therapies, training of existing models is still necessary before landing to the clinical field. For instance, even efficient tools such as AlphaFold are able to predict protein folding but still fail in RNA structure prediction, because the molecular constraints followed by RNA molecules are not fully known yet [166]. Similarly, host-microbe interactions are based on a code that is far from being deciphered, highlighting the need to persist with a wet approach. Nevertheless, in parallel with technical advances in AI-based algorithms, we recently started to collect results of clinical trials using drugs predicted by such tools. Moreover, advancements in imaging methodologies applicable in vivo will be crucial to overcome present constraints in identifying the precise sub-compartment localisation of live microorganisms in skin layers. This will enable the field to better understand the distinct crosstalk occurring in different epithelial niches, validate interactions between the cutaneous microbiome and stem or differentiated keratinocytes, and provide a clearer identification of endogenous intracellular bacteria. Of course, although high-demanding in terms of resources, longitudinal studies with multiple time points will also be important to highlight specific stages of functional/dysfunctional host-microbe interactions and the dynamic evolution of the microbiome upon therapeutic interventions.

## GLOSSARY

MICROBIOME: The ensemble of microorganisms existing in a tissue/organ. They include bacteria, viruses, fungi and other mites, but also their genetic and metabolic products.
INTER-KINGDOM (or CROSS-KINGDOM) INTERACTION: The crosstalk between organisms belonging to distinct taxonomic groups. This involves multiple types of organisms at the same time (e.g. mammal host-fungus-virus).
INTERACTOME: The whole set of functional interactions within a cell or organism (or between host and its microbiome).
META-OMICS: The analyses of the whole genetic material (metagenomics), RNA (metatranscriptomics), proteins (metaproteomics), metabolites (metabolomics) of an entire biological community or environment, rather than focusing on a single selected organism.
NETWORK INTEGRATION: A computational method to analyze and represent the interactions within complex systems, where each object is modeled as a node and interactions are depicted as arrows.
FUNCTIONAL CROSSTALK: Moving beyond descriptive compositional data, it refers to the biological roles of molecular and metabolic interactions between microorganisms.
DYSBIOSIS: Any imbalance in the composition of homeostatic microbial communities. It is characterised by potentially harmful microbes taking over commensal ones, sometimes leading to pathological states.
ECOLOGICAL MEMORY: The ability of an epithelial ecosystem, that includes a community of skin cells and the epithelial microbiome, to adapt to environmental stresses and remember previous exposures, in order to better cope with subsequent perturbations. Indeed, in this Review we propose that, in epithelial barriers, the concept of ecological memory includes both the epithelial memory of microbial variations and the microbial memory of epithelial cell adaptations to stimuli, representing an ecological and reciprocal adaptation through host–microbiome crosstalk.
MICROBIAL MEMORY OF EPITHELIAL CELLS: The ability of tissue-resident microbiomes to remember alterations affecting an epithelium such as gut and skin, possibly resulting in an adaptive response upon subsequent stimulations.
EPITHELIAL MEMORY OF MICROBIOME: The ability of epithelial cells in barrier tissues to acquire memory of antecedent variations of microbial communities. This ability might result in adaptive response upon subsequent perturbations.
